# Molecular Epidemiological Characteristics of *Staphylococcus pseudintermedius*, *Staphylococcus coagulans,* and Coagulase-Negative Staphylococci Cultured from Clinical Canine Skin and Ear Samples in Queensland

**DOI:** 10.3390/antibiotics14010080

**Published:** 2025-01-13

**Authors:** Sara Horsman, Julian Zaugg, Erika Meler, Deirdre Mikkelsen, Ricardo J. Soares Magalhães, Justine S. Gibson

**Affiliations:** 1 School of Veterinary Science, The University of Queensland, Gatton, QLD 4343, Australia; e.meler@uq.edu.au (E.M.); gibson.j@uq.edu.au (J.S.G.); 2 Australian Centre for Ecogenomics, School of Chemistry and Molecular Biosciences, The University of Queensland, Brisbane, QLD 4072, Australia; j.zaugg@uq.edu.au; 3 School of Agriculture and Food Sustainability, The University of Queensland, Brisbane, QLD 4072, Australia; d.mikkelsen@uq.edu.au

**Keywords:** canine skin infections, canine ear infections, whole-genome sequencing, methicillin-resistant *Staphylococcus* species, methicillin-sensitive *Staphylococcus* species, multilocus sequence typing, resistance genes, virulence genes, *Staphylococcus pseudintermedius*, *Staphylococcus coagulans*

## Abstract

**Background/Objectives:** Infections in dogs caused by methicillin-resistant staphylococci (MRS) present limited treatment options. This study’s objective was to investigate the molecular epidemiology of *Staphylococcus* spp. cultured exclusively from clinical canine skin and ear samples in Queensland, Australia, using whole-genome sequencing (WGS). **Methods:** Forty-two *Staphylococcus* spp. isolated from clinical canine skin and ear samples, from an unknown number of dogs, were sourced from two veterinary diagnostic laboratories between January 2022 and May 2023. These isolates underwent matrix-assisted laser desorption ionisation– time of flight bacterial identification, minimum inhibitory concentration testing using Sensititre^TM^ plates and WGS. Phylogenetic trees and core genome multilocus sequence typing (cgMLST) minimum spanning trees (MSTs) were constructed. **Results:** The isolates included methicillin-resistant and -sensitive *S. pseudintermedius* (MRSP: 57.1%, 24/42; and MSSP: 19.1%, 8/42), methicillin-resistant and -sensitive *S. coagulans* (MRSC: 14.3%, 6/42; and MSSC: 2.4%, 1/42) and methicillin-resistant coagulase-negative staphylococci (MR-CoNS: 7.1%, 3/42). Thirty-nine isolates were included after WGS, where all MRS harboured the *mecA* gene. Eighteen sequence types (STs) were identified, including three novel MRSP and six novel MSSP STs. MRSP ST496-V-VII (23%; 9/39) and MRSP ST749-IV-(IVg) (12.8%; 5/39) were commonly isolated. Phylogenetic analysis of single nucleotide polymorphisms showed that MRSP, MRSC and MSSC were similar to globally isolated staphylococci from canine skin and ear infections. Using cgMLST MSTs, MRSP isolates were not closely related to global strains. **Conclusions:** Our findings revealed a genotypically diverse geographical distribution and phylogenetic relatedness of staphylococci cultured from clinical canine skin and ear samples across Queensland. This highlights the importance of ongoing surveillance to aid in evidence-based treatment decisions and antimicrobial stewardship.

## 1. Introduction

*Staphylococcus pseudintermedius* is responsible for about 90% of skin infections [1,2,3,4] and between 20% and 94.3% of ear infections in dogs, globally [2,4,5,6]. Other coagulase-positive (CoPS) and -negative staphylococci (CoNS) have also been isolated from canine pyoderma and otitis externa [3,7,8,9]. *Staphylococcus coagulans*, a CoPS spp., is recognised interchangeably with *Staphylococcus aureus* as the second or third most isolated pathogen from canine pyoderma [1]. CoNS spp., such as *Staphylococcus epidermidis* and *Staphylococcus haemolyticus*, are rare pathogens and may be considered contaminants unless cultured from an intact primary skin lesion without the presence of other more pathogenic organisms [1,10].

Although CoNS spp. are considered to be less pathogenic than CoPS spp. [11], they may act as reservoirs for antimicrobial resistance and virulence genes [10,12], which can transfer between *Staphylococcus* spp. on mobile genetic elements [13]. Therefore, it is important to explore the lesser-researched *S. coagulans* and CoNS spp., in addition to identifying the more pathogenic *S. pseudintermedius* sequence types (STs). This investigation could identify similarities in resistance and virulence genes among staphylococci, contributing to a greater understanding of the pathogenesis of canine skin and ear infections [14].

Treating canine skin and ear infections caused by methicillin-resistant *Staphylococcus* (MRS) such as methicillin-resistant *S. pseudintermedius* (MRSP), methicillin-resistant *S. coagulans* (MRSC) and methicillin-resistant coagulase-negative staphylococci (MR-CoNS), is challenging [15]. These bacteria have acquired the *mecA* gene or its homolog *mecC*, which encodes a modified penicillin-binding protein (PBP2a) with low affinity to beta-lactams, reducing their efficacy [16]. MRS are classified as multidrug-resistant (MDR) and can express co-resistance to various antimicrobial drug classes [17]. In Australia, otitis externa in dogs caused by MRSP may be treated with florfenicol (used in Osurnia^®^) [18]. Pyoderma caused by MRS may be treated using third-line drugs, including marbofloxacin, pradofloxacin, rifampicin and amikacin, if resistance to first- and second-line options are identified after culture and antimicrobial susceptibility testing [18].

Certain STs, such as MRSP ST71, may harbour more resistance and virulence genes than other STs [19]. Identifying STs and the presence of genes helps us understand the genetic relatedness of MRS from canine skin and ear infections within a population. This aids in identifying novel highly resistant and virulent lineages, tracking population changes and guiding antimicrobial stewardship programmes [19].

Studies have focused on identifying multilocus sequence types (MLSTs) of MRSP and methicillin-sensitive *S. pseudintermedius* (MSSP) [20,21,22]. These studies revealed genotypic diversity in STs, of resistance and virulence genes [20,21,23], with MSSP STs being more diverse [22]. MRSP STs have been globally disseminated, with some STs being predominant in particular countries [24]. In Australia, reported STs include ST45, ST71, ST496 and ST749 [21,23], compared to, ST45, ST68, ST71, ST181, ST258 and ST496 in other countries [20,24,25,26]. Additionally, staphylococcal cassette chromosome *mec* (SCC*mec*) types II-III, IV and V have been identified from *S. pseudintermedius* [20,21,23].

*Staphylococcus coagulans* has been differentiated using *Sma*I pulsed-field gel electrophoresis (PFGE) and SCC*mec* types for MRSC, demonstrating high clonality within the species [7,27,28], particularly with SCC*mec* type IV and V [27,28]. However, *S. coagulans* currently has no available MLSTs. Furthermore, few studies have investigated the STs of *S. epidermidis* and *S. haemolyticus* isolated from dogs [29,30,31]. Further research is needed to understand the genotypic diversity in MRS and methicillin-sensitive *Staphylococcus* (MSS) STs in dogs with skin and ear infections. This is due to the potential transmission between dogs and humans, including dog owners and veterinary clinicians [29,32,33].

The objective of this study was to investigate the molecular epidemiology of MRS and MSS cultured exclusively from clinical canine skin and ear samples in Queensland, Australia, using whole-genome sequencing.

## 2. Results

### 2.1. Overall Study Population

Forty-two *Staphylococcus* spp. isolates cultured from clinical canine skin (69%; 29/42) or ear (31%; 13/42) samples were analysed (i.e., one isolate per sample). Matrix-assisted laser desorption ionisation–time of flight (MALDI-TOF) identified 32 (76.2%) isolates as *S. pseudintermedius*, seven (16.7%) as *S. schleiferi* and three (7.1%) as CoNS spp. (one each of *S. epidermidis*, *S. haemolyticus* and *S. petrasii*). No *S. aureus* isolates were stored by the diagnostic laboratories or collected during the study period.

The 42 isolates were cultured from dogs across 29 unique veterinary clinic postcodes. Eight isolates were cultured at the Veterinary Laboratory Services (VLS) from the same veterinary clinic postcode located in rural southeast Queensland (SE-QLD), referred to as Gatton, QLD, for the sampling location. The remaining 34 isolates were cultured at the Brisbane IDEXX Laboratory from dogs across 28 unique clinic postcodes (23 postcodes from urban SE-QLD, one from rural SE-QLD and four from north Queensland (N-QLD)). Most isolates were from dogs with samples submitted from urban SE-QLD clinic postcodes (69.1%; 29/42 samples), compared to rural SE-QLD postcodes, including Gatton and rural SE-QLD (21.4%; 9/42) and N-QLD (9.5%; 4/42). A higher proportion of samples were taken from males (57.1%; 24/42) compared to females (42.9%; 18/42). For all detailed demographic factors of dogs per MRS and MSS isolate, refer to Table 1.

### 2.2. Antimicrobial Susceptibility Testing Results

Based on the MALDI-TOF bacterial identification and tube coagulase tests, antimicrobial susceptibility testing identified 33 phenotypic MRS including 24 *S. pseudintermedius*, six *S. coagulans* and three CoNS spp. Additionally, nine phenotypic MSS were identified, comprising eight *S. pseudintermedius* and one *S. coagulans* (Table 1).

All MRS isolates (100%; 33/33) were resistant to multiple antimicrobial classes (Table 2) and tested positive for the *mecA* gene. All MRS were reported as resistant to all beta-lactam antimicrobials [34], but the number of isolates per MIC in the tables was stated as the original reading from the Sensititre^TM^ plates (Table 2). Two MSSP isolates were classified as MDR based on resistance to at least one beta-lactam, aminoglycoside (amikacin) and tetracyclines.

All MRSP isolates (100%; 24/24) were resistant to amikacin and tetracycline (Table 2). MRSC (100%; 6/6) and MR-CoNS spp. (100%; 3/3) were resistant to amikacin and doxycycline (Table 2). At least 70% of MRSP were resistant to eight non-beta-lactam antimicrobials, including chloramphenicol, clindamycin, doxycycline, minocycline, enrofloxacin, marbofloxacin, pradofloxacin, and erythromycin (Table 2). MRSC isolates (83.3%; 5/6) were resistant to enrofloxacin and tetracycline (Table 2). MSSP isolates (n = 8) were highly susceptible, with resistances observed for amikacin (100%), tetracycline (87.5%), doxycycline (62.5%), penicillin and ampicillin (50%), minocycline (25%) and trimethoprim-sulfamethoxazole (12.5%) (Table 2). The MSSC isolate was highly susceptible to all but three antimicrobial drugs (Table 2). Refer to Table 2 for all antimicrobial susceptibility results and Tables S1–S3 in the Supplementary File displaying the MIC results for *S. pseudintermedius*, *S. coagulans* and CoNS spp.

### 2.3. Molecular Characteristics of Methicillin-Resistant and -Sensitive Staphylococci Study Population

Overall, 92.9% (39/42) of isolates, including MRSP (95.8%; 23/24), MSSP (100%; 8/8), MRSC (83.3%; 5/6), MSSC (100%; 1/1) and MR-CoNS spp. (66.7%; 2/3), were retained in the final analyses after WGS. One isolate each of phenotypic MRSP, MRSC and MR-CoNS (originally *S. petrasii*) identified by MALDI-TOF was unclassified after sequencing due to contamination and was excluded from further analysis.

The three excluded isolates were cultured from samples submitted from urban SE-QLD clinic postcodes, resulting in 89.7% (26/29) of isolates being included from 20 postcodes in this region. All isolates were retained from Gatton, QLD (100%; 8/8), rural SE-QLD (100%; 1/1) and N-QLD (100%; 4/4), which included one postcode each for Gatton and rural SE-QLD, and four postcodes for N-QLD.

In total, 18 STs were identified by MLST in the 33 isolates for MRSP, MSSP and MR-CoNS spp., including three novel MRSP and six novel MSSP STs. For the MRS isolates, four SCC*mec* types were identified, including, III-(subtype IIIa), IV-(IVa), IV-(IVg) and V-VII. The most frequently isolated STs for MRSP were ST496-V-VII (39.1%; 9/23) and ST749-IV-(IVg) (21.7%; 5/23). One of the six *S. pseudintermedius* ST749 had no SCC*mec* identified and was classified as MSSP. One MSSP (unknown_1) had no ST or SCC*mec* identified due to contamination, but other genes were identified. Only one MRSP ST71-III-(IIIa) (4.3%; 1/23 MRSP) was identified. MR *S. epidermidis* ST640-IV-(IVa) and MR *S. haemolyticus* ST68-V-VII were detected. No STs are currently available for *S. coagulans*; however, 100% (5/5) of MRSC had SCC*mec* type V-VII.

### 2.4. Phylogenetic Analysis of Methicillin-Resistant and -Sensitive Staphylococcus pseudintermedius and Staphylococcus coagulans

**Figure 1:**
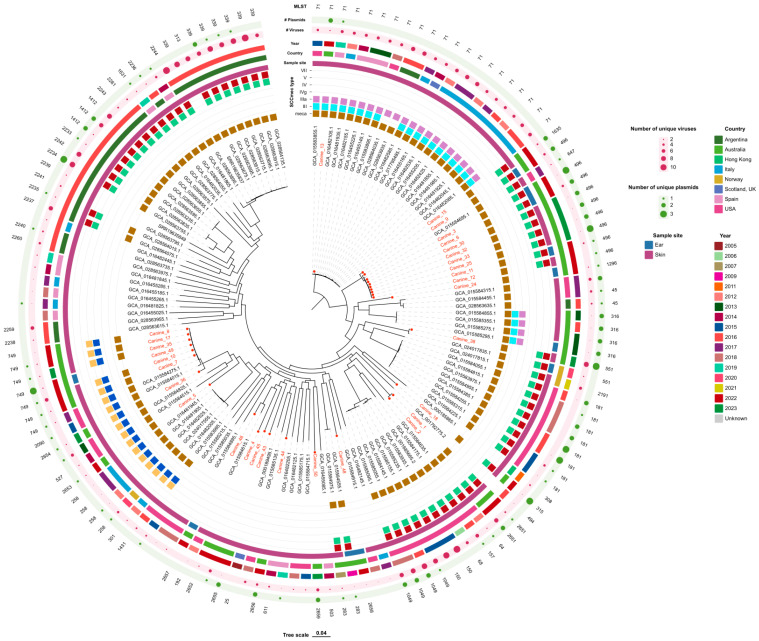
The maximum likelihood phylogenetic tree based on the core-alignment of single nucleotide polymorphisms (SNPs) of the 23 methicillin-resistant *Staphylococcus pseudintermedius* and 8 methicillin-sensitive *S. pseudintermedius* isolates cultured from clinical canine skin and ear samples. Included in the tree are 109 clinically relevant *S. pseudintermedius* genomes from the literature. The genome sequence of *S. pseudintermedius* LMG 22219 (GCA_001792775.2) was used as the reference for the SNP analysis. The isolates in this study are identified by ‘canine_#’, coloured in red. The presence of a *mecA* gene, SCC*mec* type, sample site, country and year the isolate was cultured, number of viruses and plasmids and multilocus sequence types (MLSTs) is annotated in the outer rings.

Using phylogenetic analysis based on single nucleotide polymorphisms (SNPs) revealed that MRSP ST496 isolates (canine_3, 6, 9, 11, 12, 25, 30, 32 and 33), MRSP ST1635 (canine_15) and the MRSP ST647 strain from the United States of America (USA) (accession number: GCA_015584695.1) were all closely related (Figure 1). All six ST749 clustered together, including the MSSP without a SCC*mec* (canine_7, 8, 10, 17, 35 and 49), along with MSSP ST2654 (canine_36) and two USA strains: MRSP ST257 and MRS749 (GCA_015584015.1 and GCA_015584275.1, respectively) (Figure 1). MSSP ST749 had more unique plasmids compared to the MRSP ST749 isolates. MRSP ST71 (canine_13) from this study was closely related to the global strains but had more unique plasmids and fewer viruses (Figure 1).

The two MRSP ST2651 (canine_1 and canine_2) were only closely related to each other. MRSP ST551 (canine_38) from a skin sample in our study was closely related to a MRSP ST551 cultured from a dog with otitis externa in Norway (GCA_024017835.1).

The following isolates were genetically distinct from each other, as well as from all other isolates and reference genomes in the SNP phylogenetic tree: MRSP ST315 (canine_18), MRSP ST1296 (canine_24), MSSP ST2652 (canine_4), MRSP ST2653 (canine_5), MSSP ST2655 (canine_43), MSSP ST2656 (canine_44), unknown_1 (canine_45), MSSP ST2658 (canine_48) and MSSP ST2659 (canine_50) (Figure 1).

**Figure 2:**
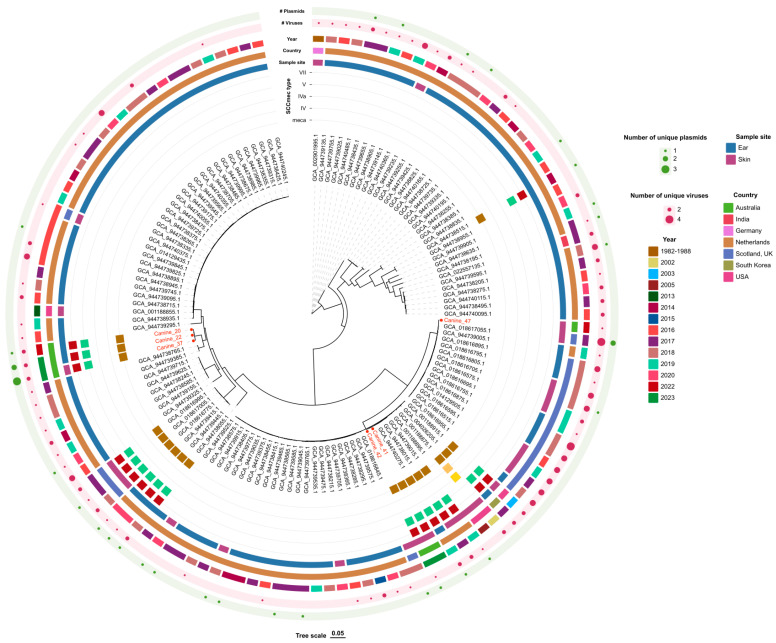
The maximum likelihood phylogenetic tree based on the core-alignment of single nucleotide polymorphisms (SNPs) of the five methicillin-resistant *Staphylococcus coagulans* and one methicillin-sensitive *S. coagulans* isolates cultured from clinical canine skin and ear samples. Included in the tree are 124 clinically relevant *S. coagulans* genomes from the literature. The genome sequence of *S. coagulans* DSM 6628 (GCA_002901995.1) was used as the reference for the SNP analysis. The isolates in this study were identified by ‘canine_#’, coloured in red. The presence of a *mecA* gene, SCC*mec* type, sample site, country and year the isolate was cultured and the number of viruses and plasmids is annotated in the outer rings.

The MSSC isolate was phylogenetically dissimilar to the MRSC isolated in this study but was closely related to other global strains (Figure 2). Of the five MRSC, three formed one cluster (canine_20, 22 and 37), while the other two formed another (canine_41 and 42) (Figure 2). Canine_41 and canine_42 MRSC isolates were phylogenetically related to other globally isolated *S. coagulans*, whereas canine_20, canine_22 and canine_37 were closely related to each other. The three clustered MRSC had the same number of unique viruses but differing numbers of unique plasmids. The MSSC had fewer unique viruses compared to globally clustered strains (Figure 2).

### 2.5. cgMLST Comparisons of Methicillin-Resistant and -Sensitive Staphylococcus pseudintermedius

**Figure 3:**
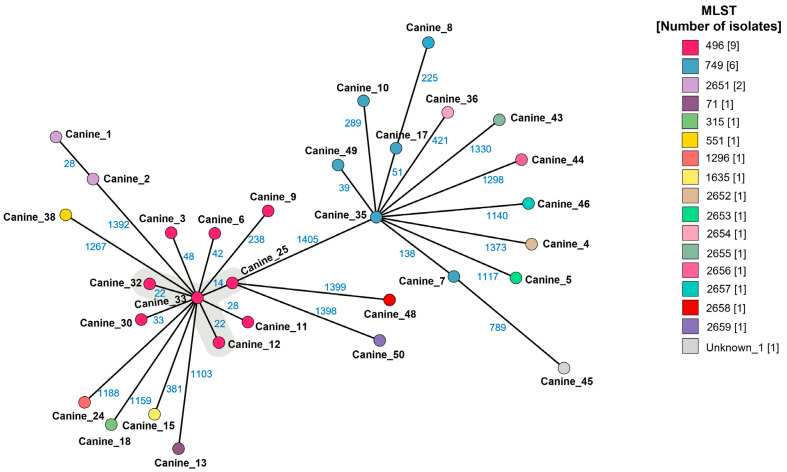
GrapeTree minimum spanning tree showing core genome multilocus sequence types (cgMLST) of the methicillin-resistant and -sensitive *Staphylococcus pseudintermedius* sequenced isolates in this study (n = 31). The name for each isolate from this study is shown next to the corresponding node or circle (canine_#). The blue numbers refer to the allelic differences between two isolates. Each node represents a unique cgMLST. Canine_49 is a MSSP ST749. Isolates with closely related genotypes (≤25 allelic differences) are shaded in grey. The lines are scaled logarithmically.

When accounting for only our study’s isolates using cgMLST minimum spanning trees, four MRSP ST496 isolates (canine_12, canine_25, canine_32 and canine_33) were found to be closely related (≤25 allelic differences) and formed a cluster (Figure 3). MRSP ST496 isolates (canine_12, canine_32 and canine_33) were isolated in urban SE-QLD, and MRSP ST496 (canine_25) was isolated from a dog from a clinic postcode in urban N-QLD. No other isolates, including MRSP ST749, were identified as being closely related (Figure 3).

**Figure 4:**
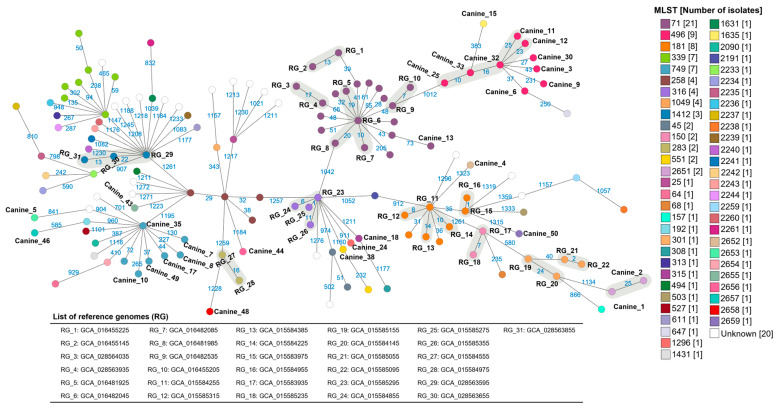
GrapeTree minimum spanning tree showing core genome multilocus sequence type (cgMLST) of the methicillin-resistant and -sensitive *Staphylococcus pseudintermedius* sequenced isolates in this study (n = 31), and the *S. pseudintermedius* reference genomes (n = 109). The name for each isolate from this study is shown next to the corresponding node or circle (canine_#). The reference genome nodes are only labelled if the isolates were closely related, denoted as RG_# (n = 31 reference genomes with ≤25 allelic differences). The list of corresponding reference genomes is in the table under the tree. The blue numbers refer to the allelic differences between genomes. Each node represents a unique cgMLST. Canine_49 is a MSSP ST749. Isolates and reference genomes with closely related genotypes (≤25 allelic differences) are shaded in grey. The lines are scaled logarithmically.

When incorporating the clinically relevant and available reference genomes, none of the isolates in this study were found to be closely related to the reference genome isolates (Figure 4). Five of the MRSP ST496 isolates (canine_11, canine_12, canine_32 and canine_33; isolated from dogs in urban SE-QLD and canine_25; dogs in urban N-QLD) and the two MRSP ST2651 isolates (canine_1 and canine_2; dogs in urban SE-QLD) formed two separate clusters when the reference genomes were included in the minimum spanning tree (Figure 4). A further twelve clusters of a total of thirty-one reference genomes were identified, including four clusters for MRSP ST71, two for MRSP ST181 and MSSP ST1049 and one each for MSSP ST150, MRSP ST283, MRSP ST316 and MRSP ST1412 (Figure 4).

### 2.6. Distribution and Comparison of the Staphylococcus spp. Phenotypic and Genotypic Characteristics

#### 2.6.1. Phenotypic and Genotypic Antimicrobial Resistance

**Figure 5:**
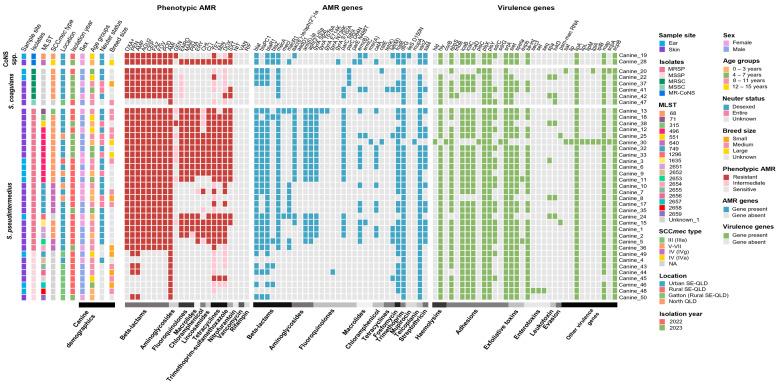
Heatmap displaying the distribution of the *Staphylococcus* spp. multilocus sequence types (MLSTs), staphylococcal cassette chromosome *mec* (SCC*mec*) types, canine demographic factors, sample site, phenotypic antimicrobial resistance (AMR) profiles and AMR and virulence genes of the *Staphylococcus* spp. isolates (ordered from top to bottom by species and MLST: two methicillin-resistant coagulase-negative staphylococci (MR-CoNS), five methicillin-resistant *S. coagulans* (MRSC), one methicillin-sensitive *S. coagulans* (MSSC), 23 methicillin-resistant *S. pseudintermedius* (MRSP), and eight methicillin-sensitive *S. pseudintermedius* (MSSP) isolates). Mutations in the fluoroquinolone and mupirocin genes were also identified as an amino acid substitution. The presence and absence of the genes (or mutations) or elements is represented by the coloured and grey blocks, respectively. The horizontal colour bar on the bottom from left to right represents the canine demographic data, antimicrobials/antimicrobial classes for the phenotypic AMR profiles, AMR genes and specific virulence factors. SE-QLD = southeast Queensland; QLD = Queensland; NA = not applicable.

Twenty-seven resistance genes, including mutations, were identified. All thirty MRS harboured the *mecA* gene, while four MRSP and two MSSP harboured the *blaZ* gene (Figure 5). Resistance genes identified in at least 50% of MRS included aminoglycosides (*aac*(6′)-*Ie*/*aph*(2″)-*Ia*, *ant*(6)-*Ia* and *aph*(3′)-*IIIa*), macrolides (*ermB*), fosfomycin (*fosB***)**, trimethoprim (*dfrG*), teicoplanin (*vanZ*) and streptothricin (*sat4*) (Figure 5). Mutations encoding known resistances to fluoroquinolones in the *gyrA*, *parC* and *parE* genes were identified in the MRS isolates. No mutations were detected in the *gyrB* gene. The most common mutations were *gyrA* S84L in 53.3% (16/30) of MRS isolates and *parC* S80I in 60% (18/30) of MRS isolates. The MR *S. haemolyticus* was the only isolate with a mutation in the *ileS* gene (D150N; mupirocin) (Figure 5). MRSC, MSSC and MSSP were highly sensitive based on MIC results, displaying fewer resistance genes compared to MRSP and MR-CoNS spp. (Figure 5).

#### 2.6.2. Virulence Genes

A total of 34 virulence genes were identified across the staphylococci isolates. The genes *plsX* and *plsY*, *srtA*, *scpB* and *lipA* were present in all isolates (100%; 39/39), with *map* (97.4%; 38/39), *hly* and *siet* (94.9%; 37/39), *ebpS*, *icaB*, *icaC* and *icaD* (87.2% 34/39) and *se*-*int* and *speta* (79.5%; 31/39) also being commonly identified.

The 23 MRSP isolates had 11 to 18 virulence genes, with 47.8% (11/23) having 15 genes. MSSP isolates had 13 to 17 genes present, with 62.5% (5/8) having 14 genes (Figure 5). MRSP ST496 had 14 to 18 genes, while virulence genes sequenced in the 5 MRSP ST749 varied, with MSSP ST749 having the same number of genes as 60% (3/5) of the MRSP isolates. The 5 MRSC isolates had unique profiles, ranging from 11 to 15 genes each. The MSSC had ten virulence genes. The MR *S. epidermidis* ST640 had 14 genes, and MR *S. haemolyticus* ST68 had 15 genes (Figure 5).

#### 2.6.3. Efflux Pumps

**Figure 6:**
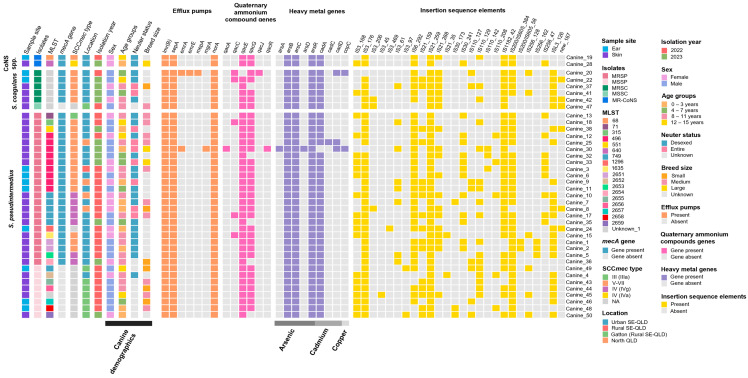
Heatmap displaying the distribution of the *Staphylococcus* spp. multilocus sequence types (MLSTs), presence of the *mecA* gene, staphylococcal cassette chromosome *mec* (SCC*mec*) types, canine demographic factors, sample site, efflux pumps, quaternary ammonium compound, heavy metal genes and insertion sequence elements with the cluster identification of the *Staphylococcus* spp. isolates (ordered from top to bottom by species and MLST: two methicillin-resistant coagulase-negative staphylococci (MR-CoNS) isolates, five methicillin-resistant *S. coagulans* (MRSC), one methicillin-sensitive *S. coagulans* (MSSC), 23 methicillin-resistant *S. pseudintermedius* (MRSP), eight methicillin-sensitive *S. pseudintermedius* (MSSP) isolates). The presence and absence of the genes or elements is represented by the coloured and grey blocks, respectively. The horizontal colour bar on the bottom from left to right represents the canine demographic data and specific heavy metals including arsenic, cadmium and copper. SE-QLD = southeast Queensland; QLD = Queensland; NA = not applicable.

The *lmr(B)*, *sepA* and *norA* efflux pumps were present in all isolates (Figure 6). A MRSP ST496 isolate (canine_30) had an additional two efflux pumps, *emrA* and *mgrA*. One MRSC isolate had an additional three efflux pumps, *emrA*, *emrE* and *mepA* (Figure 6).

#### 2.6.4. Quaternary Ammonium Compound Genes

All isolates had at least one *qac* gene (Figure 6). Most isolates had *qacE* (97.4%; 38/39) and *qacG* (87.2%; 34/39), while *qacC* was present in 12.8% (5/39) (Figure 6). Only one MRSP isolate had *qacA* and *qacR*, and one MRSC isolate had *qacJ*. The majority of MRSP isolates (69.6%; 16/23) had two *qac* genes, with one MRSP ST496 having four *qac* genes (canine_30). All but one MSSP isolate had two *qac* genes (*qacE* and *qacG*). Among MRSC, two ear isolates had three *qac* genes, with 33.3% (1/3) of the skin isolates having only one *qac* gene and 66.7% (2/3) having two. The MSSC isolate and MR-CoNS spp. isolates had two *qac* genes, *qacE* and *qacG* (Figure 6).

#### 2.6.5. Heavy Metal Genes

All isolates had the *arsB*, *arsC* and *arsR* genes, encoding resistance to arsenic (Figure 6). One MRSP ST496 isolate (canine_30) also had the *arsA* and *arsD* genes. All but one isolate had the *cadA* gene, encoding resistance to cadmium, with *cadA*, *cadC* and *cadD* being present in another MRSP ST496 isolate (canine_25). The *cadD* gene was present in one MRSP and one MRSC isolate. The *copC* gene, encoding copper resistance, was present in one MRSP and one MRSC isolate (Figure 6).

#### 2.6.6. Insertion Sequence Elements

Ten insertion sequence (IS) elements were identified with varying numbers of clusters (Figure 6). The most common IS families were IS*21* (100%; 39/39), IS*3* (97.4%; 38/39), IS*200*/IS*605* (89.7%; 35/39), IS*6* (84.6%; 33/39), IS*1182* (84.6%; 33/39), IS*110* (26.7%; 26/39), ISL*3* (61.5%; 24/39), IS*256* (48.7%; 19/39), IS*30* (41%; 16/39) and IS new_*167* (15.4%; 6/39). IS*256* was present in 10.3% (4/39) of isolates from the ears, including two MRSP and one each for MSSP and MRSC, and 38.5% (15/39) from the skin, including eleven MRSP, two MSSP, one MRSC and the MR *S. epidermidis*.

All MRSP isolates had at least one IS*3* and IS*21* cluster (100%; 23/23 isolates), with IS*6* present in 95.6% (22/23), IS*1182* and IS*200*/IS*605* in 91.3% (21/23), IS*110* in 60.9% (14/23), IS*30* and IS*256* in 56.4% (13/23), ISL*3* in 52.2% (12/23) and IS new_*167* present in 13% (3/23) (Figure 6). All MSSP isolates had at least one IS*3*, IS*21*, IS*110*, or IS*200*/IS*605* (100%; 8/8), whereas 75% (6/8) had ISL*3*, 50% had IS*6* and IS*1182* (4/8), 37.5% had IS*256* (3/8) and 25% isolates had IS*30* (2/8). IS*6*, IS*21* and ISL*3* were present in all MRSC isolates (100%; 5/5). The MSSC isolate had five IS elements, and both MR-CoNS spp. had ISE*3*, IS*6*, IS*1182* and IS*200*/IS*605*, with varying numbers of IS clusters (Figure 6).

## 3. Discussion

This study is the first to investigate the molecular epidemiology of MRS and MSS including *S. pseudintermedius*, *S. coagulans* and CoNS spp. from canine skin and ear infections. By including other commonly isolated staphylococci beyond *S. pseudintermedius*, we gained insights into the diverse genotypic landscape of these isolates, which were cultured exclusively from clinical skin and ear samples in dogs in Queensland, Australia.

In this study, MRSP ST496 and ST749, previously identified in Queensland, Australia, were prevalent [21,23]. Rynhoud et al. [23] identified these STs and showed that they were common in colonising and clinical strains in SE-QLD dogs from 2003 to 2017. Our study expands on this, indicating that clinical MRSP ST496 and MRSP ST749 may be more widespread, as they were isolated from dogs in N-QLD. Their widespread distribution, coupled with high resistance and virulence, highlights the need for implementing large-scale ongoing surveillance throughout Queensland to further comprehend the geographical distribution and detect changes over time.

Our study’s only MRSP ST71 isolate showed a close genetic relationship to global MRSP ST71 strains based on SNP phylogenetic analysis. However, this strain has been more commonly reported in Europe and North America [24,35,36], with previous research identifying either no MRSP ST71 [21] or low numbers (7.4%; 6/81 MRSP strains) [23] in Queensland animals. Unfortunately, in this study, the dogs’ historical data from which this MRSP ST71 isolate was cultured were unavailable, making it unclear if the dog had travelled to Queensland from another country or location in Australia. Despite its limited isolation suggesting a minor role in canine skin and ear infections in Queensland, larger scale studies are necessary for validation.

Although considered secondary pathogens and less pathogenic in veterinary medicine, MR-CoNS spp. are noteworthy. The MR *S. epidermidis* ST640 identified in this study was isolated from humans with urinary tract and bloodstream infections worldwide [37,38,39]. Hence, further investigations into the prevalence and pathogenic potential of MR-CoNS spp. isolated from canine skin and ear infections are necessary, as even secondary pathogens may pose a health risk.

The MRSP ST749 isolates in this study, unlike MRSP ST496, had no resistance genes/mutations to aminoglycosides and fluoroquinolones and were phenotypically sensitive to gentamicin, enrofloxacin and marbofloxacin, which are recommended, respectively, as topical or systemic treatments for skin and ear infections in dogs [40,41,42]. Similarly to Rynhoud et al. [23], MRSP ST496 had a higher proportion of antimicrobial resistance genes overall. Additionally, four of the nine MRSP ST496 isolates cultured in our study were closely related according to cgMLST, whereas all nine were closely related based on pairwise distances using SNPs. In contrast, the MRSP ST749 isolates showed higher allelic differences and were not closely related according to cgMLST. However, in terms of SNP pairwise distances, all ST749 isolates were closely related. Thus, as closely related MRSP ST496 and MRSP ST749 isolates were found in both SE-QLD and N-QLD, this study highlights the potential for clonal spread throughout QLD that should be investigated further.

Two of the three novel MRSP STs, including ST2651 and ST2653, were highly resistant. The two novel MRSP ST2651, isolated from unique dogs based on differences in the available demographic factors, were closely related using cgMLST when including the reference genomes in the minimum spanning tree. This indicated that MRSP ST2651 may be a potential emerging ST, requiring further investigations into its possible relevance in canine skin and ear infections. Additionally, MRSC had fewer antimicrobial resistance genes than most MRSP isolates and were susceptible to clindamycin, cephalothin, marbofloxacin and gentamicin, commonly used to treat skin and ear infections in dogs [40,41,42]. Thus, treating MRSP ST749 and MRSC infections may result in a lower risk of treatment failure, given their susceptibility to empirical antimicrobial therapies [40,41,42]. Due to the highly resistant MRSP and MR-CoNS spp. identified in this study, ongoing surveillance is crucial. Monitoring previously reported and emerging STs, assessing changes in prominent STs and understanding their resistance and virulence genes are imperative for the effective management of these infections.

Heavy metal resistance genes to arsenic and cadmium, and quaternary ammonium compound (*qac*) were present in all isolates, while the copper resistance gene (*copC*) was only present in one MRSP ST496 isolate. These genes likely emerge due to selective pressures from the widespread use of disinfectants or co-selection, enabling staphylococcal strains to persist in the environment and disseminate antimicrobial resistance genes [43,44]. *Qac* genes increase biocide tolerance for various antiseptics and disinfectants, along with the *norA* and *sepA* efflux pumps, in *Staphylococcus* spp. [45,46,47,48]. *QacA*- and *qacJ*-positive strains have a higher tolerance for biocides, compared to *qacB* and *qacG*, respectively [45,46,47]. The *qacA* gene, a member of the major facilitator superfamily, is regulated by the repressor protein *qacR*, both present in only one MRSP ST496 isolate [49,50], whereas *qacC*, *qacE*, *qacG* and *qacJ* are members of the small multidrug resistance (SMR) family, with *qacE* and *qacG* being identified in at least 87.2% (34/39) of isolates [49,51]. The SMR family can reduce susceptibility to quaternary ammonium compounds but does not result in “resistance” [51]. Worthing et al. [52] identified that *qac* genes found in veterinary-related MRSP did not affect biocide tolerance when there was no presence of organic matter, but the efficacy was significantly affected when organic matter was present. Given the presence of the *qac* genes and efflux pumps, veterinarians should continue to use appropriate cleaning practices prior to disinfection including, for instance, quaternary ammonium disinfectants or accelerated hydrogen peroxide, to aid in infection control and reduce the risk of potential zoonotic transmission [1].

Insertion sequences (ISs) are diverse transposable elements found in chromosomes, plasmids and SCC*mec* elements [53]. They facilitate the movement of genes, including antimicrobial resistance genes, across the genome and between bacterial populations, enabling horizontal gene transfer [54]. The IS*21* family, a widespread transposable element, was previously identified in methicillin-resistant *S. aureus* [55], and was found in all isolates in this study. The IS*6*, present in 84.6% (33/39) of our isolates, has been associated with genetic rearrangement and the spread of clinically relevant antimicrobial resistance [56]. IS*256* was previously identified in *S. pseudintermedius* isolated from canine pyoderma and was suggested to play a role in the dissemination of antimicrobial resistance [57]. IS*256* was also found in human nosocomial *S. epidermidis*, associated with biofilm formation, the *icaADBC* operon and gentamicin and oxacillin resistance [39]. To our knowledge, this is the first study to report the presence of IS*256* in MRSP, MSSP, MRSC and MR *S. epidermidis* cultured exclusively from clinical canine skin and ear samples. This emphasises the importance of further exploring ISs in staphylococcal isolates cultured from canine skin and ear infections.

This study has several limitations. Firstly, the number of collected staphylococci isolates was low, as it relied on veterinarians submitting samples to the diagnostic laboratories and was also a convenience sampling study. This resulted in 18 unique STs, which was insufficient for robust statistical comparisons involving canine demographic and genotypic factors. Veterinarians may have only submitted samples for culture and antimicrobial susceptibility testing when empirical antimicrobial treatment failed or new skin lesions appeared after treatment [10,58,59]. This might also partly explain why 76.2% (32/42) of isolates were cultured from dogs aged ≥4 years, as they may have had prior antimicrobial exposure resulting in resistance, leading to treatment failure and requiring culture and antimicrobial susceptibility testing. Secondly, the reason for submitting samples to the laboratories may have differed, resulting in a population difference in cultured isolates. For instance, VLS had all MSS isolates from a single veterinary clinic postcode, while the Brisbane IDEXX laboratory primarily had MRS from 28 clinic postcodes. This variability, along with the potential for selective sample submission by veterinarians, likely resulted in low sample size and selection bias towards MRS, limiting the representativeness of the isolates throughout QLD. Thirdly, three isolates were excluded due to contamination, whilst a MSSP isolate lacked an assigned ST but was included in the remaining analyses. Fourthly, global ST comparisons relied on available sequence data from clinically relevant studies in repositories. Sequence types may not have been included if they lacked sufficient metadata to determine animal species, sample site and clinical versus colonising strains. This limited the ability to compare the isolates in our study with other global populations, along with the two Australian-based studies due to a lack of metadata [21] and strains not being clinically relevant for the focus of this study [23]. Lastly, as there were only two CoNS spp., no phylogenetic analyses were conducted for those isolates, limiting the ability to infer genetic relationships and epidemiological patterns.

Future studies should aim to collect MRS and MSS from clinical canine skin and ear samples from all available veterinary diagnostic laboratories throughout Australia. While Worthing et al. [21] explored the clonal diversity and geographic distribution of clinical MRSP cultured from Australian animals, the study did not compare sample sites. This limitation hindered conclusions about MRSP from potential skin and ear samples and other *Staphylococcus* spp. as the study solely focused on *S. pseudintermedius* [21]. A larger study, including all staphylococci isolates cultured from clinical canine skin and ear samples, would enable comparisons of genotypic diversity among MRS and MSS across different Australian states and territories. Additionally, further research is needed to investigate genotypic similarities and the potential for zoonotic transmission of MRS and MSS from clinical canine skin and ear samples. This could be achieved by collecting isolates cultured from humans (i.e., dog owners and those with infections) and dogs with skin and ear infections. This investigation is important considering their potential to cause infections in both animals and humans, along with a potential One Health significance, which may currently be unreported [37,38,39].

This study revealed the geographical distribution of genotypically diverse staphylococci cultured from clinical canine skin and ear samples across Queensland. By identifying globally reported MRSP and novel MRSP and MSSP STs, this study offers insights that may aid in the clinical management of dogs with staphylococcal skin and ear infections. Additionally, the phylogenetic relatedness using SNPs of internationally reported strains demonstrates the interconnectedness of staphylococci cultured from canine skin and ear infections worldwide. Ongoing surveillance of MRS and MSS to comprehend the genotypic diversity of clinically significant staphylococci from dogs with these infections is important and would aid evidence-based treatment decisions and contribute to effective antimicrobial stewardship.

## 4. Materials and Methods

### 4.1. Study Population

Methicillin-resistant and -sensitive *Staphylococcus* isolates cultured from suspect clinical canine skin and ear samples were collected from two veterinary diagnostic laboratories in Queensland between 7 January 2022 and 5 May 2023. One isolate per sample, deemed clinically relevant by the laboratories, was collected using convenience sampling. The laboratories included IDEXX Laboratory (IDEXX Laboratories Pty. Ltd., Brisbane, Queensland, Australia) in Brisbane, Queensland and the University of Queensland’s Veterinary Laboratory Services (VLS) in Gatton. Samples originated from urban and rural areas in southeast Queensland (SE-QLD) and north Queensland (N-QLD).

Information extracted from the veterinary laboratory systems included submission number, sampled body site, clinic location (i.e., the location of sampling), canine demographic data including animal species, sex (male and female), age (age groups: 0–3 years, 4–7 years, 8–11 years and 12–15 years), neuter status (entire, desexed and unknown) and breed (breed size: small, medium, large and unknown) and bacterial culture results following the collection of all isolates. As these isolates were collected from diagnostic laboratories, the associated dogs’ clinical and historical data were unavailable, and it is unknown whether any samples were submitted from the same dog more than once during the study period. Refer to Table S4 for the metadata for the 39 isolates included in the WGS analysis.

### 4.2. Laboratory Sample Processing

Standard diagnostic tests were performed on the samples submitted to the veterinary diagnostic laboratories. At the Brisbane IDEXX laboratory, the skin samples were cultured on Columbia horse blood/MacConkey (HBA/MAC), Columbia horse blood with colistin and nalidixic acid agar plates (CNA), along with anaerobic with nalidixic acid agar plates (ANA and ANA/NALI) if required. The ear samples were inoculated on Columbia horse blood with colistin and nalidixic acid/MacConkey agar (CNA/MAC), CNA, Columbia horse blood agar plates (HBA) and ANA/NALI if required. Suspect *Staphylococcus* spp. were Gram-stained, and the organisms were identified using matrix-assisted laser desorption ionisation–time of flight mass spectrometry (MALDI-TOF MS; Bruker Corporation, Bremen, Germany). Antimicrobial susceptibility testing was conducted using Vitek^®^ 2 Compact (bioMérieux, Marcy-l’Étoile, France).

At VLS, samples were cultured on sheep blood agar (SBA), MacConkey agar and Sabouraud dextrose agar with chloramphenicol. *Staphylococcus* spp. were identified using a Gram-stain, catalase test, Microbact Staphylococccal 12S identification system, DNAse and tube coagulase tests. Antimicrobial susceptibility testing was conducted using disc diffusion, with methicillin resistance tested if the isolate was resistant to beta-lactam antimicrobials.

The isolates were stored on Mueller–Hinton plates in the refrigerator at 4 °C at the Brisbane veterinary diagnostic laboratory prior to collection. Upon collection, isolates were transported to VLS in a cooler box at 4 °C. The VLS-collected isolates were stored in a brain heart infusion (BHI) with 20% glycerol and retrieved from the −80 °C freezer. All suscept *Staphylococcus* spp. isolates were plated onto SBA and incubated at 37 °C for 18–24 h. The isolates were replated on SBA and incubated at 37 °C for 18–24 h to ensure pure culture. All suspect *Staphylococcus* spp. isolates were stored in the BHI with 20% glycerol at −80 °C for further microbiological and molecular testing.

### 4.3. Bacterial Identification

To confirm identification, all isolates were cultured overnight on SBA to obtain isolated colonies that underwent MALDI-TOF MS (Bruker Corporation, Bremen, Germany) at the Department of Agriculture and Fisheries Biosecurity Sciences Laboratory, Coopers Plains, Queensland, Australia, as previously described by Horsman et al. [60]. For isolates identified as *S. schleiferi* via MALDI-TOF, tube coagulase tests were conducted to identify suspect subspecies before sequencing. Briefly, two to three colonies from SBA incubated at 37 °C for 24 h were suspended in 500 µL rabbit plasma. The suspension was incubated at 37 °C for 4 h and observed for the presence of clotting. The isolates were incubated again at 37 °C for final observation at 24 h to confirm the negative results. *S. aureus* ATCC^®^29213 and *S. epidermidis* ATCC^®^12228 were the positive- and negative-coagulase control strains, respectively.

### 4.4. Antimicrobial Susceptibility Testing

Antimicrobial susceptibility testing (AST) using minimum inhibitory concentrations (MICs) was performed for the identified *Staphylococcus* spp. isolates using the Sensititre^TM^ Companion Animal Gram Positive Vet AST Plates (COMPGP1F; Thermo Fisher Scientific, Melbourne, Victoria, Australia) at VLS. *Staphylococcus* spp. isolates from −80 °C were plated on SBA and incubated at 37 °C for 24 h. Next, 50 µL sterile Mueller–Hinton broth (T3462; Thermo Fisher Scientific, Melbourne, Victoria, Australia) was pipetted into the negative control well. Three to five colonies of the organism were suspended in 5 mL sterile saline to measure 0.5 McFarland Standard manually. This bacterial suspension (30 µL) was pipetted into 11 mL Mueller–Hinton broth and vortexed for 30 s. The Sensititre^TM^ AST plate was loaded with 50 µL of the suspension per well, except the negative control, sealed and then incubated aerobically at 37 °C for 24 h to ensure detection of oxacillin resistance. After incubation, the results were manually interpreted to visualise growth (turbidity).

The isolates were tested against 24 antimicrobials. Chloramphenicol, erythromycin, gentamicin, nitrofurantoin, oxacillin + 2% NaCl, penicillin, rifampin, trimethoprim-sulfamethoxazole and vancomycin were interpreted using the M100 (human-specific) Clinical and Laboratory Standards Institute (CLSI) guidelines released in 2022 (32nd Edition) [61]. Amikacin, amoxicillin-clavulanate, ampicillin, cefazolin, cefovecin, cefpodoxime, cephalothin, clindamycin, doxycycline, enrofloxacin, marbofloxacin, minocycline, pradofloxacin and tetracycline were interpreted using the VETS01S (animal-specific) CLSI guidelines released in 2020 (5th Edition) [62]. To identify phenotypic methicillin resistance, oxacillin + 2% NaCl was used. Imipenem was not analysed as no interpretation criteria were available. *S. aureus* ATCC^®^43300 and *S. aureus* ATCC^®^29213 were the positive and negative *mecA* control strains for MIC, respectively. All MRS that were confirmed as *mecA*-positive after whole-genome sequencing (WGS) were reported as resistant to all tested beta-lactam antimicrobials [34].

### 4.5. Molecular Characterisation

#### 4.5.1. DNA Extraction and Whole-Genome Sequencing

Forty-two *Staphylococcus* spp. isolates that were collected from the two diagnostic laboratories in this study were incubated on SBA at 37 °C for 24 h. Individual colonies were used to the inoculate sterile microcentrifuge tubes of 1 mL of nutrient broth, which was then incubated at 37 °C for 24 h. After incubation, the tubes were centrifuged for 5 min at 8117× *g* to pellet the cells. The supernatant was discarded, and the pellets were stored at −80 °C for further analysis.

The pelleted *Staphylococcus* spp. isolates were sent to the Australian Centre for Ecogenomics (ACE; the University of Queensland, St Lucia, Queensland, Australia) at 4 °C in one batch for DNA extraction using the PowerSoil QiaCube HT Kit and QiaCube high throughput robot (QIAGEN; Venlo, The Netherlands) and sequenced to a depth of 1 Gbp. Libraries were prepared by ACE according to the manufacturer’s protocol for using the Nextera DNA Prep Library Preparation Kit (Illumina # 20060059; Illumina, San Diego, CA, USA). The libraries underwent quality control, and were pooled and prepared for sequencing on the NovaSeq6000 (Illumina, San Diego, CA, USA) using NovaSeq6000 SP Kit version 1.5 based on a 2 × 150 bp paired end chemistry.

#### 4.5.2. Genome Assembly, Protein Prediction and Annotation

Raw reads for the isolated staphylococci were quality-filtered with Trimmomatic (version 0.39; ILLUMINACLIP: NexteraPE-PE.fa:2:30:10, SLIDINGWINDOW:4:15) [63]. Quality-filtered reads were assembled using SPAdes (version 3.14.0) [64] as part of the Shovill assembly pipeline (version 1.1.0, T. Seeman, unpublished, https://github.com/tseemann/shovill; accessed on 2 June 2020). The completeness and contamination of each assembly were evaluated using CheckM (version 1.1.3) [65] and taxonomy was assigned to each isolate using the Genome Taxonomy Database Toolkit (GTDB-Tk; version 2.3.0; with reference to GTDB R08-RS214) [66,67].

Multilocus sequence typing was performed with MLST (version 2.19.0, unpublished, https://github.com/tseemann/mlst; accessed on 28 February 2023) which scans against PubMLST typing schemes (https://pubmlst.org/; accessed on 28 February 2023) [68]. Nine novel *S. pseudintermedius* isolates were submitted to pubMLST to assign STs (pubMLST *S. pseudintermedius* ID: 3143 to 3151), with three housekeeping gene alleles in two isolates requiring allele allocation.

Protein-coding sequences were predicted using Pyrodigal (version 2.1.0) [69] as part of Bakta (version 1.8.1) [70], using ‘trusted’ proteins from type strains of the same species: *S. coagulans*: DSM 6628 (Genbank accession GCA_002901995.1), *S. pseudintermedius*: LMG 22219 (GCA_001792775.2), *S. haemolyticus*: ATCC 29970 (GCA_006094395.1), *S. epidermidis*: NBRC 100911 (GCA_006742205.1), *S. schleiferi*: NCTC12218 (GCA_900458895.1) and *S. delphini*: NCTC12225 (GCA_900636325.1).

Antimicrobial resistance (AMR) genes were identified with AMRFinderPlus (version 3.11.14) [71]. For the AMR genes associated with mutations, the specific gene sequences were aligned with MAFFT (version 7.455) [72] and visualised using AliView (version 1.28) [73] to detect known amino acid substitutions encoding the resistances in the literature [7,74,75,76,77,78,79,80]. Staphylococcal cassette chromosome (SCC*mec*) typing in each isolate draft genome assembly was determined with staphopia-sccmec (version 1.0.0) [81]. The presence of virulence genes was identified for each isolate using the Virulence Factor Database [82]. To confirm the presence/absence of select genes that were not reported or potentially misannotated, predicted protein-coding sequences were aligned against the corresponding reference protein sequences with DIAMOND [83]. Insertion sequence elements were identified with ISEScan [84].

A total of 237 reference genomes originally isolated from canine skin and ear samples were identified from the literature and included in the present study. These reference genomes were interrogated from GenBank (National Center for Biotechnology Information) and the European Nucleotide Archive (EMBL’s European Bioinformatics Institute). This included 124 *S. coagulans* and 109 *S. pseudintermedius* genomes*,* and one each of *S. delphini*, *S. epidermidis*, *S. haemolyticus* and *S. schleiferi*. Three of the reference genomes required assembly (no assembly file was available); this included one *S. epidermidis* (SRA run ID: SRR18493467) and two *S. pseudintermedius* (SRA run IDs: SRR19635837 and SRR19635849); these were assembled using the same methods described above for the strains. All 237 reference genomes were then processed using the same methods used for the strains. Refer to Table S5 in the Supplementary Materials for the list of reference genomes.

#### 4.5.3. Phylogenetic Analysis

A phylogenetic tree was constructed for the 31 *S. pseudintermedius* (23 MRSP and 8 MSSP) and 6 *S. coagulans* (five MRSC and one MSSC) isolate draft genomes, and 109 and 124 reference genomes, respectively, based on a core-alignment of the single nucleotide polymorphisms (SNPs) followed by the removal of recombinant regions. Genomes were first aligned against their corresponding type strain genomes, including *S. coagulans* DSM 6628 (GCA_002901995.1) and *S. pseudintermedius* LMG 22219 (GCA_001792775.2), using Parsnp (version 1.7.4) [85]. Predicted SNPs for each strain were then integrated into their respective type strain genome to generate strain-specific pseudogenomes. Recombinant regions were identified and removed from the alignment of the pseudogenomes using Gubbins (version 3.3.0) [86]. A maximum likelihood phylogenetic tree was constructed using RAxML (version 8.2.12) [87], using a general time-reversible nucleotide substitution model with gamma correction for site variation (GTRGAMMA) and 1000 bootstraps. A maximum likelihood phylogenetic tree was then constructed using RAxML [87] and visualised using tvBOT [84]. Phylogenetic trees were not constructed for the CoNS spp. as there were few isolates in this study and few available clinically relevant reference genomes. Pairwise distances between all isolates and reference genomes were calculated from the SNP trees using the ‘cophenetic.phylo’ function from the ape package [88] in R (version 4.4.0). Refer to Tables S6 and S7 in the Supplementary Materials for the pairwise SNP distances for the *S. pseudintermedius* and Tables S8 and S9 for the *S. coagulans* isolates.

#### 4.5.4. Virus and Plasmid Prediction

Viral and plasmid sequences were identified and extracted from all genomes using geNomad (version 1.7.0) [89]. Host flanking regions for predicted viral sequences were trimmed using CheckV [90]. Viral (or plasmid) sequences sharing 95% identity and 85% coverage were then clustered using rapid genome clustering based on pairwise ANI, using the script aniclust.py provided as part of CheckV.

#### 4.5.5. Core Genome Multilocus Sequence Typing (cgMLST)

To investigate the presence of clonal relationships between *S. pseudintermedius* (MRSP and MSSP) isolates collected in this study only, and being inclusive of the reference genomes, core genome multilocus sequence typing (cgMLST) was performed using chewBBACA (version 3.3.2) [91]. Firstly, Pyrodigal (version 3.3.0) was used to create a training file from the *S. pseudintermedius* type strain LMG 22219. Providing the training file and the isolates as input, the chewBBACA ‘CreateSchema’ module was then used to create a whole-genome MLST (wgMLST) schema. The resulting 3363 (isolates collected in this study only) and 3980 (isolates collected in this study and reference genomes) genes were then compared, and paralogs identified using the ‘AlleleCall’ module. Only 2 and 17 genes were identified as paralogs for the isolates collected in this study only, and isolates and reference genomes, respectively; these genes were subsequently removed with the ‘RemoveGenes’ module. Finally, the ‘ExtractCgMLST’ module was used to identify the set of loci constituting the core genome, being present in 95% of the genomes. cgMLST minimum spanning trees (MSTs) were visualised using GrapeTree, including our study’s isolates only and inclusive of the reference genomes [92]. Pairwise cgMLST distances were calculated using cgmlst-dists (version 0.4.0, https://github.com/tseemann/cgmlst-dists; accessed on 15 November 2024), with a threshold of ≤25 allelic differences used to define clusters of closely related *S. pseudintermedius* isolates [93]. Refer to Tables S10 and S11 in the Supplementary Materials for the cgMLST allelic distances for the *S. pseudintermedius* isolates collected in this study only and reference genomes.

#### 4.5.6. Distribution and Comparison of the Canine Demographic and Phenotypic and Genotypic Characteristics of *Staphylococcus* Species

To identify the distribution of the canine demographic factors and bacterial molecular characteristics of the *Staphylococcus* spp. isolates, a presence-absence matrix heatmap was created using the package ‘ComplexHeatmap’ (version 2.16.0) [94,95] in R statistical software (version 4.3.1) [96]. The data included: MLSTs and/or SCC*mec* types, sampled body site, geographical location of sampling, demographic data (sex, age range and breed size), phenotypic antimicrobial resistance using the MIC results, and other molecular characteristics including antimicrobial resistance genes or mutations, virulence, efflux pumps, heavy metal and quaternary ammonium compound genes and insertion sequence elements (family and subgroup or cluster) present in each isolate.

## Figures and Tables

**Table 1:** Demographic factors from dogs corresponding to the *Staphylococcus* species isolates identified using MALDI-TOF and antimicrobial susceptibility testing that were cultured from submitted clinical skin and ear samples in Queensland, 2022–2023.

Demographic Factor		Number of Methicillin-Resistant and -Sensitive Staphylococci Isolates					
		MRSP (N = 24)	MSSP (N = 8)	MRSC (N = 6)	MSSC (N = 1)	MR-CoNS (N = 3)	Total (N = 42)
**Sampled body site**							
Skin	n (%)	18 (75.0)	5 (62.5)	3 (50.0)	1 (100)	2 (66.7)	**29 (69.0)**
Ear	n (%)	6 (25.0)	3 (37.5)	3 (50.0)	0 (0)	1 (33.3)	**13 (31.0)**
**Sampling location**							
Urban SE-QLD	n (%)	17 (70.8)	1 (12.5)	6 (100)	0 (0)	3 (100)	**29 (69.1)**
Rural SE-QLD	n (%)	3 (12.5)	0 (0)	0 (0)	0 (0)	0 (0)	**1 (2.4)**
Gatton, QLD	n (%)	0 (0)	7 (87.5)	0 (0)	1 (100)	0 (0)	**8 (19.0)**
North QLD	n (%)	4 (16.7)	0 (0)	0 (0)	0 (0)	0 (0)	**4 (9.5)**
**Sex**							
Male	n (%)	13 (54.2)	6 (75.0)	4 (66.7)	1 (100)	0 (0)	**24 (57.1)**
Female	n (%)	11 (45.8)	2 (25.0)	2 (33.3)	0 (0)	3 (100)	**18 (42.9)**
**Neuter status**							
Entire	n (%)	8 (33.3)	0 (0)	2 (33.3)	0 (0)	0 (0)	**10 (23.8)**
Desexed	n (%)	15 (62.5)	1 (12.5)	4 (66.7)	0 (0)	3 (100)	**23 (54.8)**
Unknown	n (%)	1 (4.2)	7 (87.5)	0 (0)	1 (100)	0 (0)	**9 (21.4)**
**Age group**							
0–3 years	n (%)	7 (29.2)	2 (25.0)	0 (0)	0 (0)	1 (33.3)	**10 (23.8)**
4–7 years	n (%)	5 (20.8)	1 (12.5)	2 (33.3)	0 (0)	0 (0)	**8 (19.0)**
8–11 years	n (%)	10 (41.7)	3 (37.5)	2 (33.3)	1 (100)	1 (33.3)	**17 (40.5)**
12–15 years	n (%)	2 (8.3)	2 (25.0)	2 (33.3)	0 (0)	1 (33.3)	**7 (16.7)**
**Breed size**							
Small	n (%)	2 (8.3)	3 (37.5)	2 (33.3)	0 (0)	0 (0)	**7 (16.7)**
Medium	n (%)	7 (29.2)	3 (37.5)	0 (0)	1 (100)	0 (0)	**11 (26.2)**
Large	n (%)	2 (8.3)	0 (0)	0 (0)	0 (0)	2 (66.7)	**4 (9.5)**
Unknown	n (%)	13 (54.2)	2 (25.0)	4 (66.7)	0 (0)	1 (33.3)	**20 (47.6)**

Gatton, QLD, is located within rural southeast Queensland (SE-QLD) but was kept separate as isolates were collected from the same clinic postcode and cultured at VLS. The row headings refer to sampled body site = location on the dogs’ body from which the veterinarians sampled (either skin or ear); sampling location = veterinary clinic location in QLD where the samples were taken; sex = sex of the dogs (male or female); neuter status = whether the samples were collected from dogs that were entire, desexed or unknown (i.e., not reported); age group = the dogs ages were classified into four-year intervals; breed size = the dogs breeds were classified as either small, medium, large or unknown (i.e., not reported). N = total number of isolates; n = number of isolates per variable; % = number of isolates per variable divided by total isolates per bacterial species; MRSP = methicillin-resistant *S. pseudintermedius*; MSSP = methicillin-sensitive *S. pseudintermedius*; MRSC = methicillin-resistant *S. coagulans*; MSSC = methicillin-sensitive *S. coagulans*; MR-CoNS = methicillin-resistant coagulase negative staphylococci; SE-QLD = southeast Queensland; QLD = Queensland.

**Table 2:** Non-susceptibility to antimicrobials for the methicillin-resistant and -sensitive staphylococci canine skin and ear isolates identified using MALDI-TOF based on minimum inhibitory concentrations (MICs).

Antimicrobials	Methicillin-Resistant and -Sensitive Staphylococci Isolates Non-Sensitive to the Tested Antimicrobials (N = 42)				
	MRSP (N = 24)	MSSP (N = 8)	MRSC (N = 6)	MSSC (N = 1)	MR-CoNS (N = 3)
	n (%)	n (%)	n (%)	n (%)	n (%)
**Amikacin**	24 (100)	8 (100)	6 (100)	1 (100)	3 (100)
**Penicillin**	24 (100) ^a^	4 (50)	6 (100) ^a^	0 (0)	3 (100) ^a^
**Ampicillin**	24 (100) ^a^	4 (50)	6 (100) ^a^	0 (0)	3 (100) ^a^
**Amoxicillin/clavulanate (2:1)**	24 (100) ^a^	0 (0)	6 (100) ^a^	0 (0)	3 (100) ^a^
**Cefovecin**	24 (100) ^a^	0 (0)	6 (100) ^a^	0 (0)	3 (100) ^a^
**Cefpodoxime**	24 (100) ^a^	0 (0)	6 (100) ^a^	0 (0)	3 (100) ^a^
**Cefazolin**	24 (100) ^a^	0 (0)	6 (100) ^a^	0 (0)	3 (100) ^a^
**Cephalothin**	24 (100) ^a^	0 (0)	6 (100) ^a^	0 (0)	3 (100) ^a^
**Chloramphenicol**	18 (75)	0 (0)	1 (16.7)	0 (0)	1 (33.3)
**Clindamycin**	17 (70.8)	0 (0)	1 (16.7)	0 (0)	1 (33.3)
**Doxycycline**	22 (91.7)	5 (62.5)	6 (100)	1 (100)	3 (100)
**Minocycline**	18 (75)	2 (25)	1 (16.7)	0 (0)	1 (33.3)
**Tetracycline**	24 (100)	7 (87.5)	5 (83.3)	1 (100)	3 (100)
**Enrofloxacin**	18 (75)	0 (0)	5 (83.3)	0 (0)	1 (33.3)
**Marbofloxacin**	18 (75)	0 (0)	1 (16.7)	0 (0)	1 (33.3)
**Pradofloxacin**	18 (75)	0 (0)	1 (16.7)	0 (0)	1 (33.3)
**Erythromycin**	18 (75)	0 (0)	1 (16.7)	0 (0)	1 (33.3)
**Gentamicin**	9 (37.5)	0 (0)	1 (16.7)	0 (0)	1 (33.3)
**Imipenem**	NA	NA	NA	NA	NA
**Nitrofurantoin**	0 (0)	0 (0)	0 (0)	0 (0)	0 (0)
**Oxacillin + 2% NaCl**	24 (100)	0 (0)	6 (100)	0 (0)	3 (100)
**Rifampin**	0 (0)	0 (0)	0 (0)	0 (0)	0 (0)
**Trimethoprim-sulfamethoxazole (1:19)**	15 (62.5)	1 (12.5)	3 (50)	0 (0)	2 (66.7)
**Vancomycin**	0 (0)	0 (0)	1 (16.7)	0 (0)	0 (0)

Non-susceptible isolates included intermediate and resistant isolates. No break points were available for imipenem and could not be interpreted. MRSP = methicillin-resistant *S. pseudintermedius*; MSSP = methicillin-sensitive *S. pseudintermedius*; MRSC = methicillin-resistant *S. coagulans*; MSSC = methicillin-sensitive *S. coagulans*; MR-CoNS = methicillin-resistant coagulase negative staphylococci; N = total number of isolates; NA = not applicable. ^a^ = resistant to all beta-lactams due to harbouring the *mecA* gene.

## Data Availability

The sequencing read data and genomes for the final 39 isolates described in this study have been deposited in the NCBI Sequence Read Archive (SRA) and Genbank, respectively, under the bioproject accession PRJNA1185906. All other datasets generated during and/or analysed during the current study are not publicly available due to the data sharing consent from the laboratories, but data are available from the corresponding authors on reasonable request.

## Supplementary Materials

The following supporting information can be downloaded at: https://www.mdpi.com/article/10.3390/antibiotics14010080/s1. Figure S1: Ethics email; Table S1: *Staphylococcus pseudintermedius* MIC profiles; Table S2: *Staphylococcus coagulans* MIC profiles; Table S3: Coagulase-negative staphylococci MIC profiles within Supplementary File; Table S4: Metadata; Table S5: Reference genomes; Tables S6 and S7: *Staphylococcus pseudintermedius* pairwise distance for SNP phylogenetic analysis; Tables S8 and S9: *Staphylococcus coagulans* pairwise distance for SNP phylogenetic analysis; Tables S10 and S11: *Staphylococcus pseudintermedius* allelic distances for cgMLST.
