# Supplementary material for: Molecular Epidemiological Characteristics of Staphylococcus pseudintermedius, Staphylococcus coagulans, and Coagulase-Negative Staphylococci Cultured from Clinical Canine Skin and Ear Samples in Queensland
Source: Antibiotics (Basel). 2025 Jan 13;14(1):80. doi: 10.3390/antibiotics14010080 (PMC11761246; doi:10.3390/antibiotics14010080)
Supplement: Supplementary file 1 [file antibiotics-14-00080-s001.zip › Figure S1_Tables S1 to S3_Ethics email and MIC results.pdf]

Supplementary File

Molecular epidemiological characteristics of *Staphylococcus pseudintermedius*, *Staphylococcus coagulans* and coagulase-negative staphylococci cultured from clinical canine skin and ear samples in Queensland

Sara Horsman[1*], Julian Zaugg[2], Erika Meler[1], Deirdre Mikkelsen[3], Ricardo J. Soares Magalhães[1*], Justine S. Gibson[1]

Complete postal address(es) of affiliations

[1] School of Veterinary Science, The University of Queensland, Gatton 4343 Queensland, Australia

[2] Australian Centre for Ecogenomics, School of Chemistry & Molecular Biosciences, The University of Queensland, St Lucia 4072 Queensland, Australia

[3] School of Agriculture and Food Sustainability, The University of Queensland, St Lucia 4072 Queensland, Australia

RE: Full ethics enquiry: Study collecting bacterial isolates

UQR&I Animal Ethics Unit <animal.ethics@research.uq.edu.au>

Thu 14/10/2021 8:33 AM

To: Sara Horsman <sara.horsman@uq.net.au>

Hi Sara

Thank you for contacting us regarding this. I can confirm that animal ethics approval is only required for the use of live animals (legislative) or collection of samples (UQ), it is not required where the samples to be used were not collected for the purpose of your project.

Kind Regards

Virginia

Dr Virginia Sheppard

BVSc(Hons) GPCertFelP

Coordinator, Animal Ethics and Executive Officer to AEC

Research Ethics and Integrity
The University of Queensland
Brisbane Qld 4072 Australia

T +61 7 334 68710
E v.sheppard@uq.edu.au W research.uq.edu.au

CRICOS code: 00025B

Figure S1 Response from the Animal Ethics and Executive Officer to the Animal Ethics Committee regarding whether animal ethics was required to collect staphylococci isolates and obtaining retrospective data for this study.

Table S1 Non-susceptibility and minimum inhibitory concentrations (MICs) of antimicrobials for methicillin-resistant and -sensitive *Staphylococcus pseudintermedius* clinical skin and ear isolates cultured from dogs

**Antimicrobials**	**Number of *S. pseudintermedius* isolates in each MIC (µg/mL)**											**Non-sensitive MRSP (N = 24)**	**Non-sensitive MSSP (N = 8)**
	**0.06**	**0.12**	**0.25**	**0.5**	**1**	**2**	**4**	**8**	**16**	**32**	**64**	**n (%)**	**n (%)**
**Amikacin**									32			24 (100)	8 (100)
**Penicillin**	3	1						2	26			24 (100)[a]	4 (50)
**Ampicillin**			4	1	1	2		3	21			24 (100)[a]	4 (50)
**Amoxicillin/clavulanate (2:1)**			8	2	2	3	4	3	10			24 (100)[a]	0 (0)
**Cefovecin**	1		6	1			1	3	20			24 (100)[a]	0 (0)
**Cefpodoxime**						8	2	2	20			24 (100)[a]	0 (0)
**Cefazolin**						15		17				24 (100)[a]	0 (0)
**Cephalothin**						15		17				24 (100)[a]	0 (0)
**Chloramphenicol**								14	5	1	12	18 (75)	0 (0)
**Clindamycin**				15				17				17 (70.8)	0 (0)
**Doxycycline**		5	5	2	20							22 (91.7)	5 (62.5)
**Minocycline**				12	1	2	17					18 (75)	2 (25)
**Tetracycline**			1	9	2	20						24 (100)	7 (87.5)
**Enrofloxacin**			13	1				18				18 (75)	0 (0)
**Marbofloxacin**					14		18					18 (75)	0 (0)
**Pradofloxacin**			14		1	3	14					18 (75)	0 (0)
**Erythromycin**			3	11		1		17				18 (75)	0 (0)
**Gentamicin**							23	8	1			9 (37.5)	0 (0)
**Imipenem**												NA	NA
**Nitrofurantoin**									31	1		0 (0)	0 (0)
**Oxacillin + 2% NaCl**			8		1	2	21					24 (100)	0 (0)
**Rifampin**					32							0 (0)	0 (0)
**Trimethoprim-sulfamethoxazole (1:19)**						16	6	10				15 (62.5)	1 (12.5)
**Vancomycin**					32							0 (0)	0 (0)

The white areas display the tested ranges not included in the test panels per antimicrobial agent. The grey shaded areas are the tested ranges per antimicrobial agent (light grey = susceptible, middle grey = intermediate and dark grey = resistant isolates). Where growth was present in all tested concentrations, the next serially higher concentration was given. Amikacin breakpoint is ≤4 to ≥16 µg, however, only 16 and 32 µg were tested. MIC values for amoxicillin/clavulanate (2:1; break point = 0.25/0.12-8/4) and trimethoprim-sulfamethoxazole (1:19; breakpoint = 2/38-4/76) are expressed as the MIC values for amoxicillin and trimethoprim, respectively. Non-susceptible methicillin-resistant and -sensitive *S. pseudintermedius* (MRSP and MSSP) included intermediate and resistant isolates. No break points were available for imipenem. NA = not applicable.
[a] = resistant to all beta-lactams due to harbouring the *mecA* gene.

Table S2 Non-susceptibility and minimum inhibitory concentrations (MICs) of antimicrobials for methicillin-resistant and methicillin-sensitive *Staphylococcus coagulans* clinical skin and ear isolates cultured from dogs

**Antimicrobials**	**Number of *S. coagulans* isolates in each MIC (μg/mL)**											**Non-sensitive MRSC (N = 6)**	**Non-sensitive MSSC (N = 1)**
	**0.06**	**0.12**	**0.25**	**0.5**	**1**	**2**	**4**	**8**	**16**	**32**	**64**	**n (%)**	**n (%)**
**Amikacin**									7			6 (100)	1 (100)
**Penicillin**	1		2						4			6 (100)[a]	0 (0)
**Ampicillin**			2	2			1		2			6 (100)[a]	0 (0)
**Amoxicillin/clavulanate (2:1)**			1	5		1						6 (100)[a]	0 (0)
**Cefovecin**		1			1		3	2				6 (100)[a]	0 (0)
**Cefpodoxime**						2	1	1	3			6 (100)[a]	0 (0)
**Cefazolin**						6	1					6 (100)[a]	0 (0)
**Cephalothin**						7						6 (100)[a]	0 (0)
**Chloramphenicol**								6			1	1 (16.7)	0 (0)
**Clindamycin**				6				1				1 (16.7)	0 (0)
**Doxycycline**			5		2							6 (100)	1 (100)
**Minocycline**				6			1					1 (16.7)	0 (0)
**Tetracycline**			1	4	1	1						5 (83.3)	1 (100)
**Enrofloxacin**			1	1	4			1				5 (83.3)	0 (0)
**Marbofloxacin**					6			1				1 (16.7)	0 (0)
**Pradofloxacin**			6				1					1 (16.7)	0 (0)
**Erythromycin**			3	3				1				1 (16.7)	0 (0)
**Gentamicin**							6	1				1 (16.7)	0 (0)
**Imipenem**												NA	NA
**Nitrofurantoin**									7			0 (0)	0 (0)
**Oxacillin + 2% NaCl**			1		3	1	2					6 (100)	0 (0)
**Rifampin**					7							0 (0)	0 (0)
**Trimethoprim-sulfamethoxazole (1:19)**						4	2		1			3 (50)	0 (0)
**Vancomycin**					6					1		1 (16.7)	0 (0)

The white areas display the tested ranges not included in the test panels per antimicrobial agent. The grey shaded areas are the tested ranges per antimicrobial agent (light grey = susceptible, middle grey = intermediate and dark grey = resistant isolates). Where growth was present in all tested concentrations, the next serially higher concentration was given. Amikacin breakpoint is ≤4 to ≥16 μg, however, only 16 and 32 μg were tested. MIC values for amoxicillin/clavulanate (2:1; break point = 0.25/0.12-8/4) and trimethoprim-sulfamethoxazole (1:19; breakpoint = 2/38-4/76) are expressed as the MIC values for amoxicillin and trimethoprim, respectively. Non-susceptible methicillin-resistant and -sensitive *S. coagulans* (MRSC and MSSC) included intermediate and resistant isolates. No break points were available for imipenem. NA = not applicable. [a] = resistant to all beta-lactams due to harbouring the *mecA* gene.

Table S3 Non-susceptibility and minimum inhibitory concentrations (MICs) of antimicrobials for methicillin-resistant coagulase-negative *Staphylococcus* spp. clinical skin and ear isolates cultured from dogs

Antimicrobials	Number of coagulase-negative staphylococci isolates in each MIC (µg/mL)											Non-sensitive MR-CoNS (N = 3)
	0.06	0.12	0.25	0.5	1	2	4	8	16	32	64	n (%)
**Amikacin**									3			3 (100)
**Penicillin**									3			3 (100)[a]
**Ampicillin**								2	1			3 (100)[a]
**Amoxicillin/clavulanate (2:1)**					1	1			1			3 (100)[a]
**Cefovecin**								1	2			3 (100)[a]
**Cefpodoxime**								1	2			3 (100)[a]
**Cefazolin**						1	1	1				3 (100)[a]
**Cephalothin**						2		1				3 (100)[a]
**Chloramphenicol**								2			1	1 (33.3)
**Clindamycin**				2				1				1 (33.3)
**Doxycycline**			1	1	1							3 (100)
**Minocycline**				2			1					1 (33.3)
**Tetracycline**					2	1						3 (100)
**Enrofloxacin**			2					1				1 (33.3)
**Marbofloxacin**					2			1				1 (33.3)
**Pradofloxacin**			2				1					1 (33.3)
**Erythromycin**				2				1				1 (33.3)
**Gentamicin**							2			1		1 (33.3)
**Imipenem**												NA
**Nitrofurantoin**									3			0 (0)
**Oxacillin + 2% NaCl**						1	2					3 (100)
**Rifampin**					3							0 (0)
**Trimethoprim-sulfamethoxazole (1:19)**						1		2				2 (66.7)
**Vancomycin**					3							0 (0)

The white areas display the tested ranges not included in the test panels per antimicrobial agent. The grey shaded areas are the tested ranges per antimicrobial agent (light grey = susceptible, middle grey = intermediate and dark grey = resistant isolates). Where growth was present in all tested concentrations, the next serially higher concentration was given. Amikacin breakpoint is ≤4 to ≥16 µg, however, only 16 and 32 µg were tested. MIC values for amoxicillin/clavulanate (2:1; break point = 0.25/0.12-8/4) and trimethoprim-sulfamethoxazole (1:19; breakpoint = 2/38-4/76) are expressed as the MIC values for amoxicillin and trimethoprim, respectively. Non-susceptible methicillin-resistant staphylococci (MR-CoNS) included intermediate and resistant isolates. No break points were available for imipenem. NA = not applicable. [a] = resistant to all beta-lactams due to harbouring the *mecA* gene.
